# Modelling the Extraction of Pectin towards the Valorisation of Watermelon Rind Waste

**DOI:** 10.3390/foods10040738

**Published:** 2021-03-31

**Authors:** Daniel Alexander Méndez, María José Fabra, Laura Gómez-Mascaraque, Amparo López-Rubio, Antonio Martinez-Abad

**Affiliations:** 1 Food Safety and Preservation Department, Institute of Agrochemistry and Food Technology (IATA-CSIC), 46980 Valencia, Spain; daamendezre@iata.csic.es (D.A.M.); mjfabra@iata.csic.es (M.J.F.); amparo.lopez@iata.csic.es (A.L.-R.); 2 Interdisciplinary Platform for Sustainable Plastics towards a Circular Economy—Spanish National Research Council (SusPlast-CSIC), 28006 Madrid, Spain; 3 Department of Food Chemistry & Technology, Teagasc Food Research Centre, Moorepark, Fermoy, Co., P61 C996 Cork, Ireland; laura.mascaraque@teagasc.ie

**Keywords:** pectin composition, Box–Behnken design, *Citrullus lanatus*, carbohydrate analysis, valorisation

## Abstract

Watermelon is the second largest fruit crop worldwide, with great potential to valorise its rind waste. An experimental design was used to model how extraction parameters (temperature, pH, and time) impact on the efficiency of the process, purity, esterification degree, monosaccharide composition and molar mass of watermelon rind pectin (WRP), with an insight on changes in their structural properties (linearity, branching degree and extraction severity). The models for all responses were accurately fitted (R^2^ > 90%, lack of fit *p* ≥ 0.05) and experimentally validated. At optimum yield conditions, WRP yield (13.4%), purity (540 µg/g galacturonic acid) and molar mass (106.1 kDa) were comparable to traditional pectin sources but showed a higher branching degree with longer galactan side chains and a higher protein interaction. Harsher conditions (pH 1) generated purer homogalacturonan fractions with average molar masses (80 kDa) at the expense of yield, while mild extraction conditions (pH ≥ 2) produced highly branched entangled pectin structures. This study underlines novel compositional features in WRP and the possibility of producing novel customized pectin ingredients with a wider potential application scope depending on the targeted structure.

## 1. Introduction

Around 60% of the total food waste is attributed to agricultural, post-harvesting, processing or distribution, causing significant losses for producers as well as food security and environmental concerns [1]. Watermelon (*Citrullus lanatus*) is a fruit with a great economic importance, being the second world’s largest fruit crop, with a global production of approximately 103 million tonnes in 2018 [2]. Watermelon rind (WR), which constitutes approximately 30% of the whole fruit, is most often dumped arbitrarily into the environment, raising environmental concerns [3]. The valorisation of this waste is limited due the lack of knowledge on possible conversion strategies of potential valuable compounds [4,5].

Pectin is a complex cell wall polysaccharide from plant sources, consisting mainly of homogalacturonan (HG), rhamnogalacturonan-I (RG-I) and minor rhamnogalacturonan-II (RG-II) and xylogalacturonan (XGA) regions. HG is a homopolymer consisting of α-(1–4)-linked-D-galacturonic acid units (GalA), which can be naturally methylesterified at the C-6 carboxyl group [4]. The degree of esterification (DE) governs much of the properties in pectin, where high methoxy (HM) pectin (DE 50–80%) form gels at low pH (2.5–3.5) or in the presence of high amounts of soluble solids such as sucrose (55%) and low methoxy (LM) pectin (DE < 50%) can form gels in the presence of divalent cations, and is also more resistant to a wider range of pH than HM pectin [6]. Rhamnogalacturonan-I (RGI) is generally composed of a backbone of the repeating disaccharide [→2)-α-l-rhamnopyranose-(1→4)-α-d-GalA-(1→/], sometimes substituted at the O-4 position of the rhamnose residue with neutral sugar side chains of galactose, arabinose, of varying length and composition, producing the so-called “hairy” region with RG-II [7].

Pectin has been recognized for its numerous uses as a technological adjuvant in the food industry, as its different structures may lead to different gelling, stabilization, emulsifying properties or sustained release functionalities in complex food matrices [8,9]. These properties differ depending on the complexity of the pectin structure, which in turn depend on the source and the extraction parameters. For instance, the presence of branching structures and their entanglement cause an increasing shear rate dependence of viscosity [10,11]. In addition, intermolecular forces and the nature of junction zones at which the polymer molecules are cross-linked, improves the maintenance of network together to form gels with components like divalent cations [11]. Apart from their technological value, the interest in pectins associated with human health benefits has been recently increasing, linked to the effect against diverticular disorders, diabetes, regulation of serum cholesterol and glucose levels, immunological and anticancer properties, also ultimately related to compositional and structural features in pectin [8,12].

Currently, sugar beet pulp, citrus albedo and apple pomace are the most common sources used for pectin extraction [4,13]. However, due to the high demand in the global market and growing interest in valorising side streams to obtain pectins with diverse functional properties, research attention has been given to the composition and extraction from other non-commercial sources, such as broccoli, tomato, carrots [14], onion hulls, sour cabbage, sour cucumber, endive roots or pumpkin waste, among many others [4,9]. The interest in pectin extraction from WR was originally evaluated by Campbell (2006), who investigated the effect of pH, solid-to-liquid ratio and different enzyme treatments, amongst others, in pectin yield, degree of methoxylation and galacturonic acid degree. Subsequently, the use of a weak organic and strong acids [15,16] or the effect of microwave-assisted extraction on pectin yields have also been explored [3,17], although previous works did not include a proper pectin characterization. Petkowicz, Vriesmann and Williams (2017) [18] demonstrated the functional properties of WR pectin using conventional acid extraction, but the structural and functional characterization corresponded to a specific extraction condition (0.1 N nitric acid, boiling under reflux during 1 h using a solid-liquid ratio of 1:25 (*w*/*v*)). Thus, there is a lack of information about pectin in WR and how the extraction parameters affect its composition and structure, information that is of great interest as it will ultimately affect its potential industrial uses.

This study contributes to expand this knowledge with a more comprehensive characterization of WRP, including response surface methodologies to relate how different process parameters (pH, temperature and time) affect these compositional and structural features and optimize process efficiency (pectin yield and purity) to critically discuss valorisation strategies for this waste.

## 2. Materials and Methods

### 2.1. Sample Preparation

Fresh commercial state watermelon (*Citrullus lanatus*) fruits were kindly supplied by Anecoop S. Coop. during the summer season of 2019 from Almeria, Spain. The fruits were processed, removing the red part of the fruit and keeping the white rinds, which were chopped into pieces of 0.5–2.5 cm and immersed in 4 volumes of distilled water for 10 min with gentle agitation. After that, the water was drained, and the rinds were freeze-dried. The dried material was milled in liquid nitrogen using an A11 Basic IKA mill (IKA, Staufen, Germany) and stored in desiccators at 0% humidity until used. Citrus and apple pectin (CP and AP, respectively) were used for comparative purposes (Sigma-Aldrich, Stenheim, Germany). Phenol red (ACS grade) and sodium hydroxide (pharma grade) were supplied by Sigma-Aldrich (Stenheim, Germany). Acetone (99%) was from WVR chemicals. N-hexane (>95% purity) and ethanol (96% *v*/*v*, USP grade) were purchased from Panreac Applichem (Darmstadt, Germany). Analytical standards: D-(+)-Galactose CAS:59-23-4, D-(+)-Galacturonic Acid monohydrate CAS:91510-62-2, L-Rhamnose monohydrate CAS: 10030-85-0, D-Glucoronic Acid CAS:6556-12-3, D-(+)-Mannose CAS: 3458-28-4, D-(+)-Glucose CAS: 50-99-7, D-(-)-Fructose CAS: 57-48-7, L-(-)-Fucose CAS: 2438-80-4, D-(+)-Xylose CAS:58-86-6, L-(+)-Arabinose CAS: 5328-37-0 were purchased from (Sigma-Aldrich).

### 2.2. Proximate Analysis

A proximate analysis was made to the watermelon rind. Moisture was determined according to an approved method 935.29 [19]. Protein determination was carried out by the Dumas combustion method according to ISO/TS, 16634-2 (2016) and a nitrogen conversion factor of 5.35 [20]. Lipid was measured after Soxhlet extraction according to [21]. Ash content was determined according to the standard method TAPPI T211 om-07. Carbohydrates were calculated as the sum of all sugar constituents following the method described in Section 2.6. All analyses were done in triplicate.

### 2.3. Experimental Design

The individual and interactive effects of process variables (temperature (70–95 °C, X_1_), time (30–90 min; X_2_) and pH (1–3; X_3_)) over pectin extracted from WR were evaluated. A Box–Behnken response surface experimental design (BBD) with three factors at three levels (Table 1) was chosen, the upper and lower values selected based on previous literature [22]. The experiments were developed in a randomized order with an empirical second-order regression polynomial mathematical model, which exhibits the relationship between response and independent variables. Fifteen experiments were generated with three replications at the centre points (Cep). The generation of the experimental design, analysis of variance (ANOVA), quality of fit (coefficient of determination (R^2^)), lack of fit and yield optimization were attained using the software Statgraphics^®^ Centurion, version XVIII (Manugistic, Inc., Rockville, MD, USA). After optimization, validation experiments in triplicate were performed under optimal conditions and compared with predicted values from the models.

### 2.4. Pectin Extraction

Pectin extractions were made with a solid–liquid ratio of 1:20 (*w*/*v*) in acidic aqueous solutions, adjusting the pH with a 1 M HCl solution, then heated on a hotplate with magnetic stirring for the selected time. After that, the pectin-based solution was first filtered with a muslin cloth, followed by vacuum filtration using Whatman filter paper n° 4 at 60 °C. The pectin-based solution was mixed with 96% (*v*/*v*) ethanol at a ratio of 1:2 (*v*/*v*) and left overnight in the freezer. The coagulated pectin was centrifuged at (23,450× *g* for 20 min) and washed with 96% (*v*/*v*) ethanol and acetone in consecutive washing cycles, in order to remove water and low molecular weight or polar compounds. The wet pectin was left to dry at 60 °C in a hot air oven until constant weight, then grounded and stored in a desiccator until further analysis. Pectin yield (Y%) was calculated from the following equation:

(1) $$Y(\%)=\frac{m_{0}}{m}\times 100$$

where $m_{0}$ (g) is the weight of dry pectin and m (g) is the weight of dry watermelon rind powder.

### 2.5. Determination of Pectin Composition with Titrimetric Methods

Methoxyl content (MeO%), anhydrouronic acid (AUA%) and degree of esterification (DE) of extracted pectins were measured according to the method described by Grassino [23]. Pectin samples of 0.05 g were weighed and wetted with 0.5 mL of ethanol (96%) and dissolved in 10 mL of a 10% (*w*/*v*) sodium chloride solution overnight with magnetic stirring. Two drops of phenol red were added to the dissolved pectin as indicator and the solution was titrated with 0.1 M NaOH. Then, 25 mL of 0.25 M NaOH was added and mixed vigorously to de-esterify pectin and left at room temperature for 30 min. Next, a volume of 25 mL of standardized 0.25 M HCl was added to neutralize NaOH and was titrated again with 0.1 M NaOH until the colour changed. The determinations were performed in triplicate, and citrus and apple pectin were used as references.

### 2.6. Monosaccharide Composition

The sugar composition of the extracts was determined after acidic methanolysis as previously described [24]. Freeze-dried samples (1 mg) were incubated with 1 mL of 2 M HCl in dry methanol for 5 h at 100 °C. Samples were then neutralized with pyridine, dried under a stream of air, and further hydrolysed with 2 M trifluoroacetic acid (TFA) at 120 °C for 1 h. The samples were again dried under a stream of air and re-suspended in 1mL milliQ water, filtered (0.22 µm pore size) and injected. The monosaccharides were analysed using high-performance anion exchange chromatography with pulsed amperometric detection (HPAEC-PAD) with an ICS-3000 system (Dionex, Sunnyvale, CA, USA) equipped with a CarboPac PA1 column (4 × 250 mm^2^, Dionex). Ten microliters of sample was injected and eluted at a flow rate of 1 mL/min and 30 °C. Neutral sugars were eluted in water for 16 min with post-column addition of 0.5 mL/min of 300 mM sodium hydroxide after a preconditioning isocratic step with 260 mM sodium hydroxide and 68 mM sodium acetate (7 min) and 5 min equilibration time in water prior to injection. Uronic acids were eluted in the same run with a gradient to 250 mM sodium acetate and 250 mM sodium hydroxide over 15 additional minutes. Control samples of known concentrations (0–100 mg/L; detection limit 0.1 mg/L) of mixtures of glucose (Glc), fucose (Fuc), rhamnose (Rha), galactose (Gal), arabinose (Ara), xylose (Xyl), mannose (Man), galacturonic acid (GalA) and glucuronic acid (GlcA) were used for calibration, as prepared from 10 mg/mL stock solutions (kept at −20 °C until use) and treated analogously. Due the high lability of fructose (Fru) during methanolysis, the free Fru and Glc were measured using a sucrose, D-fructose and D-glucose (K-SUFRG) Assay Kit (Megazyme, Bray, Ireland), according to the manufacturer’s instructions. All measurements were carried out in triplicate.

### 2.7. Fourier Transform Infrared (FTIR)

FTIR measurements were recorded in transmission mode in a controlled chamber at 21 °C and dry air in order to avoid humidity and CO_2_ using a Cary 630 FTIR spectrometer (Agilent, Santa Clara, CA, USA). The spectra were taken at 4 cm^−1^ resolution in a wavelength range of 400–4000 cm^−1^ and averaging a minimum of 32 scans. The acquired Spectra were processed using Origin pro 2019 software (OriginLab Corporation, Northampton, MA, USA).

### 2.8. High-Performance Size Exclusion Chromatography (HPSEC)

The molar mass (MM) of the pectin extracts was estimated by HPSEC following a previously described method with slight modifications [25]. The HPLC system was equipped with a Waters 2695 separation module and a Waters 2414 refractive index detector (Waters, Milford, MA, USA). Then, 0.1 M NaCl (aq.) was used as the mobile phase [26]. The samples (1 mg/mL) were dissolved in the mobile phase under magnetic stirring at 40 °C, filtered through 0.8 μm pore syringe filters and injected into an OHpak SB-806 HQ (8 mm × 300 mm) SEC column (Shodex, Tokio, Japan) equilibrated at 40 °C. The injection volume was 20 μL and the flow rate was 0.5 mL/min. Calibration was performed using P-82 pullulan standards (Shodex, Tokio, Japan). A deconvolution analysis was made to estimate polydispersity index (PDI). The data were processed and the peaks deconvoluted using Origin pro 2019 software (OriginLab Corporation, Northampton, MA, USA).

## 3. Results and Discussion

### 3.1. Proximate Analysis

The composition of watermelon rind (WR) was first analysed in order to assess its valorisation potential and evaluate the extraction efficiency of different components compared to the starting material. The moisture content of fresh WR (93.3%) was in agreement with similar studies [27]. Ash content was slightly lower than previously reported values [27,28], while protein content was slightly higher (Table 2). These differences might arise from different degrees of ripeness, specific cultivar used, crop condition or method of analysis employed [27,28]. The main carbohydrate constituents in WR were pectin, cellulose and free sugars (Fru and Glc). Pectin, as the sum of GalA, Rha, Gal, Ara and Fuc, accounted for around 35%, which is higher than previously reported values of 13% [29] and 19–21% [28]. Although GalA contents is an internationally recognized indicator for pectin contents, other pectin structural neutral sugars, such as the contribution of arabinogalactan can highly differ between pectin sources. These differences are actually very significant in WRP, with >10 wt% galactose in the starting material. Pectin composition was therefore mainly ascribed to galacturonan and galactan, with minor fractions of arabinan. The low Rha to Galactose content suggests longer branching side chains in RG compared to other pectin sources [14,30]. This could actually be positive, as quality criteria of pectin are ascribed to its rheological properties, linked not only to increased homogalacturonan yield but also reported to be increased with abundance of galactan side chains [31,32].

### 3.2. Model Design and Statistical Analysis

The solid–liquid ratio (SLR) is an important factor on pectin yield during extraction, with some authors reporting the proportional increase of pectin yield with the decrease of the solid–liquid ratio, due to higher content surface area between particles and solvent [33]. However, a significant difference on yield for SLR higher than 1:30 *w*/*v* has not been reported for pectin extraction [17,33,34]. In this study, 1:20 *w*/*v* was selected and fixed for all the treatments developed, based on the good results on pectin yield and purity from watermelon and other fruits [17,18,29,35], while minimizing solvent use.

Second-order polynomial equations including interactive and quadratic terms were employed to generate mathematical models to detect the optimum extraction conditions and express the relationship between process variables and responses. Response variables, all main significant effects and fitting parameters are shown in Table 3, and all equations are available in Supplementary Materials Table S1. The responses evaluated were selected based on the functionality they may confer to pectin. Yield is crucial as a value of extraction efficiency, while degree of esterification (DE), methoxyl content (MeO) and molar mass (MM) are related to thickening, gelling and other rheological properties [18]. Anhydrouronic acid (AUA) is recognized as a quality factor for pectin extraction, with 65% being recommended for food or pharmaceutical additives [36]. Moreover, the main carbohydrate constituents of pectin (GalA, Gal, Ara and Rha) were also selected to have an insight on structural changes due to their influence on the functional properties of pectin [37] (Table 3).

### 3.3. Effect of Extraction Parameters on WRP Composition and Its Structural Features

#### 3.3.1. Yield

The pectin yields from the different extractions were between 2.1% and 12.2% (Table 1). High yields were thus attained despite starting from a freeze-dried WR sample, which usually hinders wettability, consequently decreasing the pectin yield [18]. The high yields obtained can in fact be compared to conventional industrial processes of pectin extraction from citrus, apple and sugar beet pectin (25–30%, 4.2–19.8% and 15–30%, respectively) [4] and non-conventional pectin sources such as durian rind (2.3–9.3%) [6], pumpkin waste (7.4%), passion fruit (10–12.6%), banana peel (9%) or carrot pomace (5–15.2%) [4], or even recent WRP extraction using hydrochloric acid [16]. This evidences a promising potential of WR waste as an alternative cost-effective source of pectin.

The model generated with a lack of fit test *p* value ≥ 0.05, R^2^ (97.91) clearly shows a good correlation between the response and the independent variables. Based on the equation parameters (Table 3) and as observed in the 3D-surface plot (Figure S1a), it is evident that extractions conducted at low pH (1–2) have the highest influence on yield, followed by temperature (X_1_, *p* = 0.0012, X3, *p* = 0.0001). Interestingly, treatment time did not have a significant effect over pectin yield (*p* = 0.051).

The positive effect of a low pH is due to hydrolytic attack over the plant cell wall, releasing soluble pectin components [10]. Nevertheless, pH values lower than 1.35 coupled with high temperatures actually reduced yields, as can be noticed for the harshest condition (95 °C, 90 min, pH 1, 9.5%; Table 1). This might be explained by the degradation of polymeric pectin into small molar mass pectin for which the efficiency of alcohol precipitation is reduced [34,38]. On the other hand, increasing pH values above 1.35 gradually decreased the yield due to the inherent recalcitrance and cross-linked character of pectin components within the complex cell wall architecture [34], and aggregation of very high molar mass components slowing down pectin release [39,40].

The results also indicated that the yield of pectin increased with the effect of high temperature and low pH (Figure S1a, Table 1), probably due to increased solubility of pectin and diffusivity of the solvent into the plant tissue with increasing temperature [33]. Since time did not have a significant effect on yield (*p* = 0.051), 30 min was put forward as an effective extraction time to attain relatively high yields.

#### 3.3.2. Pectin Esterification

A quadratic polynomial regression was seen to properly fit the experimental data for DE and MeO, since the lack-of-fit test was insignificant for each of the three parameters. Likewise, R^2^ values obtained were all above 90%, pointing out a high reliability of the models. Factors that had a significant influence over both DE and MeO were, as with the yield, the pH, extraction temperature and their interactions (X_3_ X_1_) (Table 3), in agreement with similar studies on honey dew and sugar beet [30,41].

The combination of high temperature, long extraction times and a very low pH reduced the DE (Table 1, Figure S1b) due to hydrolytic de-esterification of the HG chains [6,39], but still producing HM pectins in all cases, in agreement with WRP extracted at different conditions [16,18]. Reported values for the DE vary depending on the type of hydrolytic extraction (e.g., enzymatic versus acid catalysed or severity of acid extraction), ranging between 38% and 46% [29]. Although DE and MeO are based on the same titration, the calculation of the DE indicates the ratio of esterified versus non esterified acid groups regardless of the purity in HG in the sample. A decreased yield of HG (if DE is constant) would thus only affect the MeO. The MeO content in WRP was between 4.8% and 7.9%, the higher values being similar to those present in commercial AP and CP (7.3% and 9.2%, respectively). The lowest degree of MeO (4.7%) was obtained when pectin was extracted at pH 3, for 60 min, at 70 °C (Table 1), because these mild conditions reduced the efficiency of HG extraction (GalA content 199.7 µg/mg; Table 4). On the contrary, the harshest conditions (pH 1, 90 min, at 95 °C) improved HG yield (GalA 672.5 µg/mg; yield 9.95%) but also showed low MeO value, (Table 1; Figure S1c) and the lowest DE (52.48%), again indicating extensive de-esterification [6,39]. Other authors have reported MeO values of 5.8–7.6% for tomato peel waste when using an ammonium oxalate buffer [31], and values of 2.2–6.2% for different apple pomace cultivars [42].

### 3.4. Constituent Sugar Composition

A quantitative sugar analysis was carried out in order to elucidate the composition of pectin extracted with each treatment condition. Moreover, different sugar ratios (R) were also calculated for each run based on previous literature, as to have an insight on how the extraction parameters affect the structural complexity of the pectin [14,41,43]. Table S2 in Supplementary Materials summarises ratios associated with the contribution of branching and its length (RB), the linearity of pectin (RL), the severity of the extraction (RS) or the overall rhamnogalacturonan contribution (RG). The main sugar components were GalA, Gal, Ara, Rha, Xyl and Fuc, in accordance with findings from other authors for carrot or watermelon [11,18]. Man, Glc, GlcA and Fru were not detected or only present in trace amounts (≤0.5 µg/mg). The lack of free sugars in the extracts indicates successful removal of non-pectin components [14]. The contribution of all the models generated for these sugar constituents (GalA, Fuc, Rha, Ara and Xyl) was significant with R^2^ >0.9 (Table 3) and no significant lack of fit, evidencing a very good accuracy of the mathematical models, except for Gal.

#### 3.4.1. Galacturonic Acid and Homogalacturonan Contribution

The most important sugar constituent in pectin is GalA, being crucial for the thickening or gelling properties. This feature is routinely evaluated for pectin quality as AUA through simple titration, given the lack of significant amounts of any other acid groups in pectin. In this work, both the AUA and the GalA content were compared and introduced in the models (Table 3, Equation (S6)). AUA values were slightly higher than the respective GalA values, a fact that may be attributed to the presence of other organic acids or traces of mineral acid from the treatments. The main factors that positively influenced their content were temperature (*p* = 0.0032) followed by pH, (*p* = 0.0009), the GalA content and AUA varying between 254 and 615 µg/mg and 33.5% and 69.1%, respectively (Table 1 and Table 4). The mildest conditions showed the lowest AUA and GalA values. This value is consistent with the low MeO, as noted above, supporting the presence of non-pectin components or pectin other than HG or RG. On the contrary, the harshest conditions resulted in high AUA values and higher linearity, as in commercial citrus and apple pectin (see RL ratio in Table S2). The higher purity of HG under these conditions comes at the expense of a slight decrease in the overall yield, as commented on above. Comparable values of AUA were found for dragon fruit 45.2–52.4% [44], apple pomace pectin 70.5% [42] and tomato peel waste 39.6–52.9% [9,31].

#### 3.4.2. Rhamnogalacturonan Content

Rha is mainly ascribed to the RG backbone of intercalating [→2)-α-l-Rha-(1→4)-α-d-GalA-(1→/] and its content was, therefore, similarly affected as the GalA content. Temperature (*p* = 0.017) and pH (*p* = 0.0002) produced a significant increase at harsh conditions, the overall values ranging from 5.6 to 24.6 (µg/mg) (Figure S2b). While Rha contents seems relatively low compared to traditional pectin sources (apple (AP) or citrus pectin (CP) sources, see Table 4), the ratio Rha to GalA is not that different from AP or CP. The lower relative abundance of Rha is a result of higher amounts of Gal and Ara compared to Rha, indicating the presence of longer sugar side chains in RG, as evidenced by high RB ratio (which refers to the side chain length) values compared to AP or CP (cf. Table S2 in Supplementary Materials). This evidences that the differences between WR and traditional pectin lies in the composition and extent of the RG rather than in the abundance of RG regions through the pectin backbone. These RB values are also higher than for other alternative pectin sources (3.4–6.3) [41]. Although the traditional “true” gel formation is governed by interaction between HG main chains, the presence of long galactan side chains instead of short-branched RGI regions has been reported to have a positive effect over polymer gelation [11,32]. In any case, the relative broad range of the branching degree depending on the extraction conditions (RB values 5.6 to 32.0) might be an advantage, as the gelling of pectin could take place either through the long arabinogalactan branched chains [11,32] or through the more linear non-methylesterified galacturonic acid chains [45].

#### 3.4.3. Arabinogalactan, Galactan and Arabinan Content

Ara content in WRP was significantly affected by pH, temperature, time and the interaction between temperature and pH (Table 3). Its contents were in a range from 0 to 59.9 µg/mg. Pectin extracted at pH 1 showed a significantly lower Ara concentration than that obtained at less aggressive pH 2–3 (see surface plot in Figure S2e in the Supplementary Material), and a complete degradation of the Ara moieties was observed with the harshest conditions, as indicated by the total Ara content (Table 4), as well as by the different severity factors (RS, Table S2). This is explained by the acid lability, the greatest among all detected sugars, of arabinofuranosyl units [41]. Immunostimulating, anticancer and strong prebiotic activities have been ascribed to the Ara and Gal branching moieties in pectin [12,46]. The good fitting to the model and the correlation to the extraction parameters points out its suitability to target changes in the functional properties of the pectins obtained, fostering the hydrolytic debranching of arabinan and arabinogalactan molecules. The presence of these long-branched chains in pectins has also been correlated with improved emulsifying capacity [10].

Gal was the most abundant neutral sugar found in all pectin extracts, its concentrations ranging from 88.4 to 252.8 µg/mg (Figure S2d). Compared with commercial CP and AP, higher amounts were present in most extraction runs indicating a high and long distribution of Gal side chains through WRP, as commented on above and evident from the high values of the RB ratio, related to average size of the branching side chains (see Table S2 in Supplementary Materials). Nevertheless, Gal content showed a low fitting accuracy to the experimental data, and no explicit tendency towards specific conditions was observed. A possible explanation would be the presence of both soluble galactans, not bound to the pectin backbone and easily extracted at mild conditions, as well as of galactans more tightly bound to it. This would explain the presence of significant quantities both at mild and relatively harsh conditions. The only parameter that had an almost significant effect was pH (*p* = 0.079), (Equation (S10) in the Supplementary Materials), as Gal readily degraded at very harsh conditions. This is evidenced when looking at the ratio of GalA to Gal, which steadily increased with increasing aggressive conditions, suggesting the presence of galactans is not directly linked to the pectin backbone at very mild conditions, mixed galactan and RGI at the centre points, and gradual degradation of Gal at the harshest conditions (Table 4).

#### 3.4.4. Other Pectin Components

The detection of Xyl suggests the presence of xylogalacturonan regions in pectin, also found in previous studies for watermelon fruit pectin, citrus pectin and carrot pectin [14]. An overall low Xyl content was found on all extracts (0–5.39 µg/mg), its content being mainly affected by pH, increasing when pectin was extracted at high pH (Table 4). The mild conditions would foster the preferential extraction of these easier extractable regions at the expense of low yields. However, when harsher conditions (combined low pH and high temperatures) were used (see surface plot in Figure S2a), Xyl content increased again, possibly due to the extraction of some Xyl containing non-pectic polysaccharides, such as xyloglucan [7].

Although fucose was not selected for modelling due to its very minor contents (1.8–3.5 µg/mg), it suggests the presence of a small proportion of RGII [43]. The highest concentration of Fuc was found at the centre points (Table 4). Its decrease at harsh conditions is probably related to degradation, while the reduced presence at mild conditions is probably related to the low pectin yields, with fucose being closely bound to the RGII regions on the main backbone.

### 3.5. Fourier Transform Infrared (FTIR)

**Figure 1:**
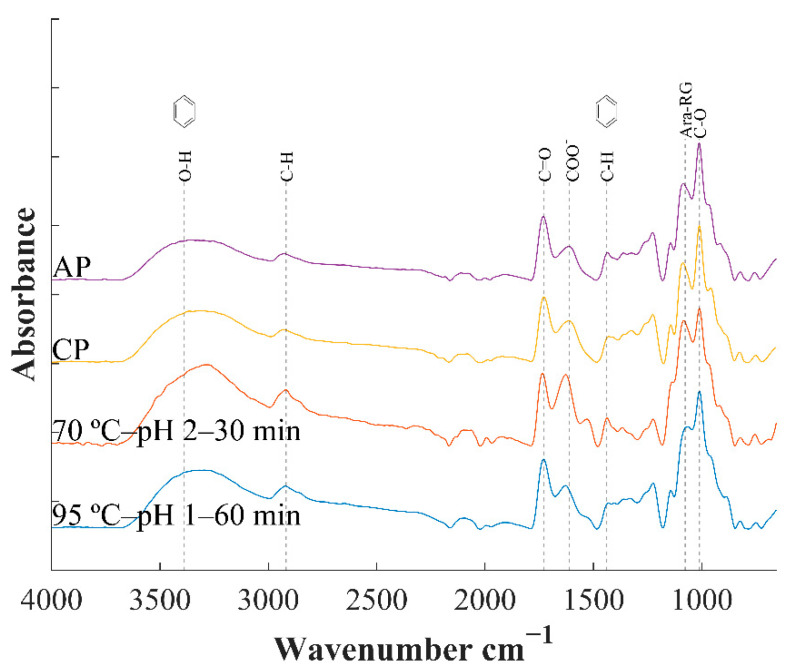
FT-IR spectra of watermelon rind pectin (WRP) extracted at softer (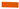) and harsh (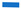) conditions in comparison with commercial apple pectin (AP) and citrus pectin (CP).

The FTIR spectrum of reference pectins (AP and CP) and two exemplary samples extracts at mild and harsh extraction conditions are presented in Figure 1. The band at 3289 cm^−1^ is mainly ascribed to the hydroxyl groups stretching of water molecules (O-H) in pectin [38], while the vibrational bands in the range 2850–2919 cm^−1^ can be attributed to the C–H of CH, CH2 and CH3 groups (Pasandide et al., 2017). The band at 1746 cm^−1^ is associated with the vibration of the esterified carbonyl group C=O and the band at 1625 cm^−1^ to the free carboxylic groups COO-, with another one at the weaker symmetric vibrating at 1428 cm^−1^ [13]. Both peaks are widely used with regards to the evaluation of the degree of esterification in pectins, which can be estimated as the ratio between the peak area of the band corresponding to the carboxylate esterified group and the sum of both mentioned bands, among other calculation methods [47,48]. However, for the present samples, peaks related to protein were identified by the weak amide II band at ~1526 cm^−^^1^ [49]. Moreover, the broad band centred around 3300 cm^−1^ also contains contributions from the amide N-H stretching (3540–3125 cm^−^^1^), which causes a sharpening of this band and further evidences the presence of proteins in the extracts. This causes the vibration band of amide I (~1655 cm^−^^1^) to overlap with the bands of interest for DE calculation, and the estimation was not viable [47]. A greater protein content is observed at mild extraction conditions compared to harsher conditions giving higher yields or to commercial pectins (CP, AP; Figure 1). Protein content from WRP extracted under optimum conditions to maximize yield (OP; Section 3.7) was indeed much higher compared to reference CP and AP pectin (8.9, 2.2 and 1.7% d.w., respectively). This points towards probable protein interaction with AG side chains in the highly branched structures, as suggested previously [50]. Although these findings could imply a lower purity of pectin for conventional uses, the presence of small amounts of protein may promote the formation of pectin complexes with interesting emulsifying properties [49,51]. Although further purification treatments to remove proteins is an option, the presence of protein could constitute a valuable factor for WRP extracts with higher versatility for diverse applications. The two strong absorption bands at 1006 and 1079 cm^−1^ are attributed to the glycosidic linkage (C-O and C-C stretching bond) and is typical for backbone vibrations of pectin [13,52]. The band at 1077 cm^−1^ can be attributed to neutral Ara-based glycans or RG regions (Kacuráková et al., 2000) and is especially patent noted at less severe (Figure 1) extraction conditions, in agreement with compositional data (Table 4) and RS values (Table S2 in Supplementary Materials).

### 3.6. Molar Mass Distribution

**Figure 2:**
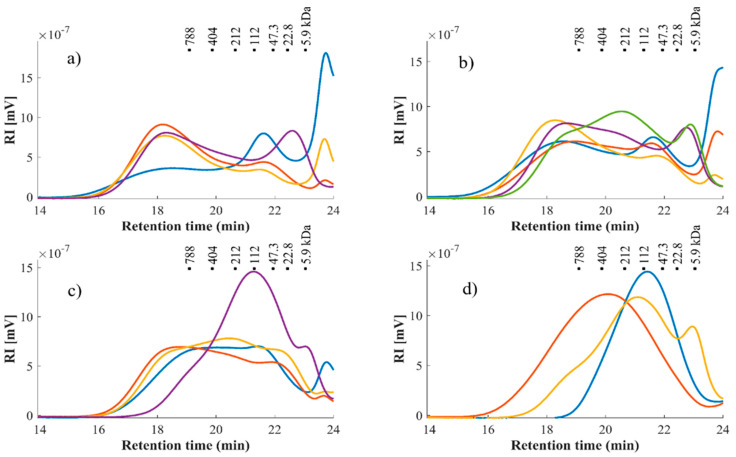
Molar mass (MM) distribution of the pectin extractions from WR studied. (**a**) 70 °C treatment runs: 6 (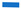), 3 (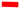), 11 (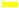), 1 (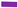). (**b**) 82.5 °C treatment runs: 4 (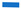), 12 (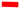), 13 (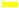), 7 (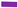), 10(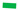). (**c**) 95 °C treatment runs: 8 (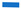), 2(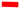), 9 (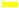), 5 (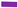). (**d**) citrus pectin (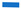), apple pectin (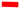) and optimum point condition pectin (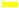). For interpretation of the numbers listed, please refer to the Table 1. The black squares correspond to pullulan standards with matching molar mass (MM).

HPSEC was used to study and obtain information about the size and distribution of molar mass (MM) from the different WRP extracts at different conditions, including the WRP sample at the optimized conditions (OP; see Section 3.7) and reference pectins (CP and AP) for comparison purposes (Figure 2).

The MM distribution of the extracts showed three main peaks, which were different in polydispersity, distribution and signal intensity, mainly depending on temperature and pH (data available in Table S3 of Supplementary Materials). Control pectins (AP, CP) showed one single peak, although AP had a broader MM distribution than CP (PDI of 8.9 and 2.0, respectively; Table S3; Figure 2). Likewise, harsh treatments displayed similar MM distribution as in reference pectins (PDI 4.0), associated with higher homogeneity and purity of these pectins. A peak in the low molecular weight region (around 5.9 kDa) was especially patent at either pH 3 or low extraction temperatures, suggesting the preferential extraction of loose or less recalcitrant pectin small fragments (Figure 2, Table S2). Under milder temperatures (e.g., pH 1, 60 min at 70 °C), higher MM and broader MM distributions (PDI 8.5) suggested the presence of highly branched pectin components, which may be forming agglomerates of different polysaccharide or protein components (Table S2). The presence of other small polydisperse components contributing to agglomeration, such as hemicelluloses, is also feasible [14,53].

For pectins extracted at 95 °C, their MM distribution presented a wider dispersity, except at pH 1. At this pH, a different peak close to 112 kDa was observed, this lower MM being similar to CP (Figure 2). This was possibly due to their less branched (or linear) homogalacturonan-based composition (Table 4, Table S2). MM for the harshest and for optimum point conditions (Section 3.7) also showed similar values (106.1 ± 2.7, 87.5 ± 0.8 kDa, respectively) (Table S4).

MM as a response factor for the mathematical model was taken from the average value of the main peak from each extract (Table S3). A very good fit was observed (Table 3), with a significant effect of all factors (time (*p* = 0.0031), temperature (*p* = 0) and pH (*p* = 0)) over MM (Figure S1d). The results again evidenced the possibility to target different structures depending on the treatment applied, with either bigger, entangled, or branched structures (higher values of MM and distribution) at mild conditions, or a linear homogeneous pectin with harsher conditions, targeting different pectin functionalities.

### 3.7. Optimization and Validation

In order to validate the model with an optimized product, the optimum conditions boosting extraction yield were generated (Table 5), resulting in a maximum yield of 13.4%, which corresponded to an extraction temperature of 95 °C, during 90 min and at pH 1.36. Three more extraction conditions and their experimental and theoretical values are presented in Supplementary Materials Table S4 for further comparison. Then, these extraction parameters were experimentally applied, and the pectin obtained at these optimum conditions (OP) was characterized for the different responses and compared with the predicted values (Table 5). In most cases, a good correlation between experimental and predicted values was obtained, thus supporting the reliability of the generated models. The amount of pectin extracted at optimum yield point was similar to previously reported results using conventional acid extraction [16,18]. OP showed overall values comparable to traditional pectin sources (CP and AP; Table 1). The MM profile in OP was also comparable to CP or AP. On the other hand, the neutral sugar composition of OP showed higher Gal contents (Table 4) and a higher RG-I contribution and branching degree compared to commercial pectins (RG-I values of 9.7, 2.8 and 2.4 for OP, AP and CP, respectively; Table S2).

## 4. Conclusions

WR waste showed relatively high pectin contents (~30%), not recalcitrant to common acid treatments (~13% yield) and increased arabinogalactan side chain contribution compared to common commercial sources like apple (AP) and citrus pectin (CP). A Box–Behnken design was used to accurately model the effect of extraction time, pH and temperature on WRP yield and composition. The broad range of extraction conditions led to pectin with a broad range of esterification degree, molar mass and compositional or structural characteristics, all of which were accurately fitted to the polynomial models. The harshest conditions generated purer homogalacturonan fractions at the expense of yield, while mild extraction conditions (pH ≥ 2) produced highly branched entangled pectin structures. Optimum yield conditions led to more linear pectins with similar molecular mass as commercial AP or CP, but with significantly higher RG-I, higher branching degree and small protein contents present, pointing towards a significant pectin–protein interaction. These unique structural features suggest that these pectins could have a better performance than commercial pectins as emulsifying agents and display a double functionality as texturizers and stabilizing agents. The study underlines novel compositional features in WRP and how they relate to extraction parameters, offering the possibility of producing novel customized pectin ingredients with a wider potential application scope depending on the targeted structure. Further studies to relate the structural characteristics with the functionality of the different pectins will be conducted to unravel the potential of this new pectin source for the production of food additives.

## Figures and Tables

**Table 1:** Box–Behnken experimental design (BBD), coded levels, and actual values (in parentheses) of the variables: Extraction temperature (X_1_, °C); time (X_2_, min); pH (X_3_). The responses considered were the yield (Y), degree of esterification (DE), anhydrouronic acid content (AUA) and methoxyl content (MeO).

	Independent Variables			Experimental Values			
Run	X_1_ (°C)	X_2_ (min)	X_3_ (pH)	Y (%)	DE (%)	AUA (%)	MeO (%)
1	−1 (70)	0 (60)	−1 (1)	6.88	65.47	59.54	6.87
2	+1 (95)	−1 (30)	0 (2)	10.72	75.30	56.92	7.55
3	−1 (70)	+1 (90)	0 (2)	7.55	76.65	55.56	7.51
4	0 (82.5)	−1 (30)	+1 (3)	2.45	87.28	43.22	6.66
5	+1 (95)	0 (60)	−1 (1)	9.95	52.48	69.08	6.39
6	−1 (70)	0 (60)	+1 (3)	2.03	81.04	33.48	4.78
7	0 (82.5)	−1 (30)	−1 (1)	8.50	62.97	66.96	7.43
8	+1 (95)	0 (60)	+1 (3)	4.44	88.25	49.70	7.73
9	+1 (95)	+1 (90)	0 (2)	12.19	76.08	57.29	7.68
10	0 (82.5)	+1 (90)	−1 (1)	10.81	58.98	66.44	6.90
11	−1 (70)	−1 (30)	0 (2)	6.92	76.85	53.60	7.26
12	0 (82.5)	+1 (90)	+1 (3)	3.44	89.05	47.91	7.03
13	0 (82.5)	0 (60)	0 (2)	8.61	78.52	61.32	8.48
14	0 (82.5)	0 (60)	0 (2)	8.04	80.88	63.16	9.00
15	0 (82.5)	0 (60)	0 (2)	7.80	80.83	54.85	7.80
OP	95	90	1.36	13.4	61.62	66.42	6.76
AP	-	-	-	-	77.18	62.17	9.00
CP	-	-	-	-	55.19	69.62	6.78

Y: yield; DE: degree of esterification; MeO: methoxyl content; AUA: anhydrouronic acid content.

**Table 2:** Composition of watermelon rind.

Proximate Analysis	Content (Dry Basis wt (%)) ^a^	Monosaccharide Composition (µg/mg Dry Basis) ^b^	
Ash	2.55 ± 0.17	GalA	167.1 ± 7.58
Fats	1.05 ± 0.15	Rha	9.2 ± 0.31
Protein	17.23 ± 0.11	Gal	111 ± 4.39
Carbohydrates	83.9 ± 3.45	Ara	17.1 ± 1.56
*of which*		Fuc	11.7 ± 0.29
*Pectin ^c^*	31.61 ± 1.4	Xyl	38.8 ± 8.85
*Free sugars ^d^*	19.47 ± 0.58	Man	9.6 ± 2.19
*Cellulose ^e^*	14.28 ± 1.4	Glu	354.93 ± 45.4
*Others ^f^*	14.21 + 1.12	Fru	120.2 ± 4.20

^a^ Based on lyophilized WR. ^b^ Monosaccharide composition determined by HPAEC-PAD. ^c^ Considered as the sum of GalA, Rha, Gal, Ara and Fuc. ^d^ Measured with D-fructose and D-glucose (K-SUFRG) Assay Kit. ^e^ Estimated as the difference between methanolysis and Saeman hydrolysis detected Glc (Saeman, 1945). ^f^ Other sugars not included in the previous groups.

**Table 3:** Main effects of process parameters ((X_1_, °C); (X_2_, min); (X_3_, pH)) and their combinations on the different responses and goodness of fit for the models.

Responses	Main Effects (*p* ≤ 0.05)	Lack of Fit (*p* ≥ 0.05)	R^2^
Y (%)	$X_{3}$ $,{\;X}_{3}^{2}$ $,\;X_{1\;}$	0.18	97.91
DE (%)	$X_{3}$ $,\;X_{1}X_{3},{\;X}_{3}^{2}$ $,\;X_{1}^{2}$	0.63	99.55
MeO (%)	$X_{3}^{2},\;X_{1}X_{3},\;X_{1}^{2}$	0.72	90.24
AUA (%)	$X_{3}$	0.54	92.62
MM (kDa)	$X_{1},$ $X_{3}^{2},$ $X_{1}^{2}$ $,\;X_{2}$ $,\;X_{1}X_{3},\;X_{1}X_{2},\;X_{2}^{2}$	0.72	99.31
GalA (μg/mg)	$X_{1},X_{3}$	0.08	94.19
Ara (μg/mg)	$X_{3},$ $X_{1},$ $X_{2}$ $,\;X_{1}X_{3}$ $,\;X_{2}^{2},$ $X_{3}^{2}$	0.31	99.60
Xyl (μg/mg)	$X_{3}$ $,\;X_{1}X_{2},$ $X_{1}X_{3},$ $X_{2}{\;X}_{3}$ $,\;X_{3}^{2}$	0.22	98.26
Rha (μg/mg)	$X_{1},\;X_{3}$	0.11	95.16
Gal (μg/mg)	$X_{3}$	-	65.76

Y: yield; DE: degree of esterification; MeO: methoxyl content; AUA: anhydrouronic acid content; MM: molar mass.

**Table 4:** Experimental values for neutral sugar (Rha, Ara, Gal and Xyl) and galacturonic acid (GalA) composition of pectins extracted according to Box–Behnken experimental design.

Run	Fucose (µg/mg)	Rha (µg/mg)	Ara (µg/mg)	Gal (µg/mg)	Xyl (µg/mg)	GalA (µg/mg)
1	3.44 ± 0.21 ^ab^	15.71 ± 0.69 ^f^	9.09 ± 0.65 ^g^	248.49 ± 12.46 ^a^	0 ± 0 ^i^	357.72 ± 15.00 ^ef^
2	3.35 ± 0.32 ^abc^	17.36 ± 1.77 ^ef^	22.44 ± 2.38 ^e^	190.9 ± 20.32 ^bc^	0.46 ± 0.80 ^hi^	442.08 ± 51.95 ^d^
3	3.30 ± 0.30 ^abcd^	16.67 ± 1.13 ^f^	31.49 ± 2.5 ^d^	162.4 ± 12.14 ^de^	0.96 ± 0.84 ^ghi^	339.07 ± 30.11 ^f^
4	2.93 ± 0.15 ^bcde^	7.45 ± 0.83 ^ij^	47.31 ± 2.49 ^b^	113.02 ± 7.68 ^g^	4.75 ± 0.49 ^b^	254.07 ± 17.53 ^g^
5	1.83 ± 0.19 ^f^	24.65 ± 1.3 ^a^	0 ± 0 ^i^	139.04 ± 9.67 ^ef^	2 ± 0.22 ^def^	615.84 ± 54.75 ^a^
6	2.84 ± 0.24 ^cde^	5.67 ± 0.41 ^j^	59.9 ± 4.11 ^a^	121.71 ± 8.44 ^fg^	4.61 ± 0.19 ^b^	199.72 ± 10.97 ^g^
7	3.34 ± 0.12 ^abc^	23.76 ± 0.63 ^ab^	5.71 ± 0.2 ^h^	252.28 ± 7.76 ^a^	0.97 ± 0.85 ^gh^	522.44 ± 3.52 ^bc^
8	2.94 ± 0.3 ^bcde^	10.43 ± 0.88 ^h^	30.4 ± 2.65 ^d^	106.42 ± 12.9 ^gh^	2.32 ± 0.29 ^cd^	368.86 ± 34.65 ^ef^
9	3.33 ± 0.28 ^abc^	19.23 ± 1.54 ^de^	18.65 ± 1.31 ^f^	203.7 ± 18.28 ^b^	1.01 ± 0.87 ^gh^	451.38 ± 39.38 ^d^
10	2.57 ± 0.3 ^e^	22.95± 1.97 ^abc^	1.7 ± 1.47 ^i^	211.81 ± 18.38 ^b^	1.05 ± 0.92 ^fgh^	517.28± 31.78 ^bc^
11	2.6 ± 0.05 ^e^	13.23 ± 0.19 ^g^	37.64 ± 0.55 ^c^	88.43 ± 1.37 ^h^	2.06 ± 0.06 ^de^	340.74 ± 18.56 ^ef^
12	2.78 ± 0.19 ^de^	8.7 ± 0.67 ^hi^	38.73 ± 2.74 ^c^	109.9 ± 11.78 ^gh^	3.07 ± 0.51 ^c^	333.7 ± 36.41 ^f^
Cep ^a^	3.54 ± 0.11 ^a^	16.7 ± 1.32 ^f^	32.82 ± 2.44 ^d^	176.57 ± 12.4 ^cd^	1.52 ± 0.53 ^efg^	386.74 ± 36.4 ^e^
OP ^b^	2.51 ± 0.99 ^e^	21.19 ± 0.85 ^cd^	2.26 ± 0.06 ^i^	204.95 ± 5.00 ^b^	1.9 ± 0.17 ^defg^	540.34 ± 37.02 ^b^
AP	1.65 ± 0 ^f^	24.19 ± 0.96 ^ab^	18.47 ± 0.58 ^f^	49.44 ± 1.68 ^i^	14.15 ± 0.76 ^a^	481.97± 17.89 ^cd^
CP	0.57 ± 0.21 ^g^	22.27 ± 0.53 ^bc^	19.46 ± 0.71 ^ef^	35.18 ± 1.59 ^i^	3.14 ± 0.1 ^c^	565.91± 22.27 ^ab^

^a^ Cep. central points. ^b^ pectin obtained at optimum yield conditions. Means followed by different letters in the same column (^a–j^) are significantly different (*p* ≤ 0.05). Glc, Fru, Man, Fuc and GlcA were not detected or only in trace amounts (<0.5 µg/mg).

**Table 5:** Comparison of experimental and predicted values of different responses parameters of WRP using optimum conditions pectin.

Responses	Experimental Value	Model Value
Y (%)	13.4 ± 0.05	13.09
DE (%)	61.55 ± 1.36	61.19
MeO (%)	6.55 ± 0.81	6.88
AUA (%)	57.88 ± 2.75	64.27
MM (kDa)	106.1 ± 2.69	* -
GalA (µg/mg)	540.34 ± 37.02	558.19
Ara (µg/mg)	2.26 ± 0.06	5.37
Fuc (µg/mg)	3.47 ± 0.23	2.23
Gal (µg/mg)	204.95 ± 5	165.78
Xyl (µg/mg)	1.71 ± 0.22	1.77
Rha (µg/mg)	21.19 ± 0.85	23.5

* MM was not calculated due to peak heterogeneity of the sample measured. Y: yield; DE: degree of esterification; MeO: methoxyl content; AUA: anhydrouronic acid content; MM: molar mass.

## Data Availability

The data presented in this study are available on request from the corresponding author.

## Supplementary Materials

The following are available online at https://www.mdpi.com/article/10.3390/foods10040738/s1, Figure S1: Response surface plot for the effects of temperature and pH with fixed time at 90 min on (a) yield (Y), (b) degree of esterification (DE), (c) methoxy content (MeO) and (d) molar mass (MM) (kDa), of pectin extracted, Figure S2: Response surface plot for the effects of temperature and pH with fixed time at 90 min on (a) xylose (Xyl), (b) rhamnose (Rha), (c) galacturonic acid (GalA), (d) galactose (Gal) and (e) arabinose (Ara) (µg/mg) content of pectin extracted., Table S1: Polynomial equations obtained for the prediction of responses properties for pectin extracted., Table S2: Composition ratios and pectin region % based on the mg/mg sample quantifiable neutral sugars and galacturonic acid, Table S3: Molar mass (MM) at distinctive highest point, peak area and polydispersity index estimated with mathematical deconvolution calculated from the HPSEC runs evaluated.
