# Supplementary material for: Modelling the Extraction of Pectin towards the Valorisation of Watermelon Rind Waste
Source: Foods. 2021 Mar 31;10(4):738. doi: 10.3390/foods10040738 (PMC8066451; doi:10.3390/foods10040738)
Supplement: Supplementary file 1 [file foods-10-00738-s001.pdf]

Supplementary Material

Modelling the extraction of pectin towards the valorisation of watermelon rind waste

Daniel Alexander Mendez[1], María José Fabra[1,2], Laura Gómez. Gómez-Mascaraque[3], Amparo López-Rubio[1,2], Antonio Martinez-Abad[1,2*]

[1] Food Safety and Preservation Department, Institute of Agrochemistry and Food Technology (IATA-CSIC), Valencia, Spain

[2] Interdisciplinary Platform for Sustainable Plastics towards a Circular Economy-Spanish National Research Council (SusPlast-CSIC), Madrid, Spain

[3] Department of Food Chemistry & Technology, Teagasc Food Research Centre, Moorepark, Fermoy, Co. Cork, Ireland

[*]Corresponding author: Antonio Martinez-Abad. E-mail address: conaba@iata.csic.es

Tel.: +34 963900022; fax: +34 963636301

Table S1. Polynomial equations obtained for the prediction of responses properties for pectin extracted.

$Y(\%)=8.81 - 0.24816X_1 - 0.11X_2 + 9.51X_3 + 0.002X_1^2 + 0.0005X_1X_2 - 0.013X_1X_3 + 0.0009X_2^2 - 0.011X_2X_3 - 2.68X_3^2$ Eq. 1

$DE(\%)=-29.90 + 2.56X_1 - 0.083X_2 - 3.16X_3 - 0.021X_1^2 + 0.0006X_1X_2 + 0.404X_1X_3 - 0.00060X_2^2 + 0.048X_2X_3 - 4.95X_3^2$ Eq. 2

$MeO(\%)=-19.99 + 0.68X_1 + 0.016X_2 - 1.32X_3 - 0.0047X_1^2 - 0.00008X_1X_2 + 0.068X_1X_3 - 0.00020X_2^2 + 0.\,0075X_2X_3 - 1.23X_3^2$ Eq. 3

$AUA(\%)=-92.90 + 3.86X_1 + 0.07X_2 - 11.51X_3 - 0.02X_1^2 - 0.0010X_1X_2 + 0.13X_1X_3 - 0.0004X_2^2 + 0.043X_2X_3 - 3.26X_3^2$ Eq. 4

$MM\ (kDa)=-23579.3 + 582.54X_1 + 106.43X_2 + 2300.88X_3 - 3.86X_1^2 - 0.86\,X_1X_2 + 16.65X_1X_3 - 0.35X_2^2 - 0.54X_2X_3 - 921.90X_3^2$ Eq. 5

$GalA\ (\mu g/mg)=-362 + 17.3X_1 - 3.53X_2 - 27\,X_3 - 0.047\,X_1^2 + 0.0155\,X_2^2 + 6.2\,X_3^2 + 0.0073\,X_1X_2 - 1.78\,X_1X_3 + 0.707\,X_2X_3$ Eq. 6

$Ara\ (\mu g/mg)= -162.3 + 2.76\,X_1 + 0.352X_2 + 80.62X_3 - 0.0158\,X_1^2 + 0.0004X_1X_2 - 0.40\,X_1X_3 - 0.0035\,X_2^2 - 0.025X_2X_3 - 6.32X_3^2$ Eq. 7

$Xyl\ (\mu g/mg)=-28.6 + 0.671X_1 - 0.1049X_2 + 5.91X_3 - 0.003X_1^2 + 0.0015X_1X_2 - 0.09X_1X_3 + 0.000013\,X_2^2 - 0.015X_2X_3 + 1.10X_3^2$ Eq. 8

$Rha\ (\mu g/mg)= -53.0 + 1.49X_1 - 0.004X_2 + 6.64X_3 - 0.0063X_1^2 - 0.0010X_1X_2 - 0.083X_1X_3 + 0.00067X_2^2 + 0.0172X_2X_3 - 1.91X_3^2$ Eq. 9

$Gal\ (\mu g/mg) = -303 + 15.5X_1 + 2.64X_2 - 203X_3 - 0.10X_1^2 + 0.0024\,X_2^2 - 5.3X_3^2 - 0.040X_1X_2 + 1.88\,X_1X_3 + 0.31X_2X_3$ Eq. 10

Table S2. Composition ratios and pectin region % based on the mg/mg sample quantifiable neutral sugars and galacturonic acid (Denman & Morris, 2015)

Run	RB [a]	RL[b]	RS [c]	HG %[d]	RG-I %[e]	HG:RG-I
1	16.39	1.30	39.34	34.20	25.79	1.32
2	12.29	1.91	19.69	42.47	21.37	1.98
3	11.62	1.60	10.76	32.23	19.42	1.66
4	21.52	1.47	5.37	24.66	16.04	1.53
5	5.64	3.71	-	59.11	13.95	4.23
6	32.01	1.04	3.33	19.40	18.17	1.06
7	10.85	1.84	91.45	49.86	25.84	1.92
8	13.11	2.46	12.13	35.84	13.70	2.61
9	11.56	1.86	24.19	43.21	22.27	1.94
10	9.30	2.17	304.89	49.43	21.39	2.31
11	9.52	2.41	9.05	32.75	12.63	2.59
12	17.09	2.08	8.61	32.50	14.88	2.18
13	12.25	1.65	13.19	40.95	23.40	1.75
14	12.72	1.63	11.38	35.32	20.48	1.72
15	12.70	1.72	10.80	34.73	19.03	1.82
OP*	9.78	2.37	238.86	51.91	20.76	2.50
AP	2.80	4.39	26.09	45.77	6.84	6.69
CP	2.45	6.98	29.08	54.36	5.50	9.86

*Pectin extracted at optimum yield conditions

[a] A larger value is indicative of larger average size of the branching side chains. (Gal+Ara/Rha).

[b] A larger value suggests of more linear/less branched pectins. (GalA/(Xyl+Rha+Ara+Gal).

[c] A larger value is indicative of more severe extraction conditions and loss of arabinofuranosyl (Araf) residues. (GalA/Ara).

[d] (GalA-Rha).

[e] (2Rha+Ara+Gal).

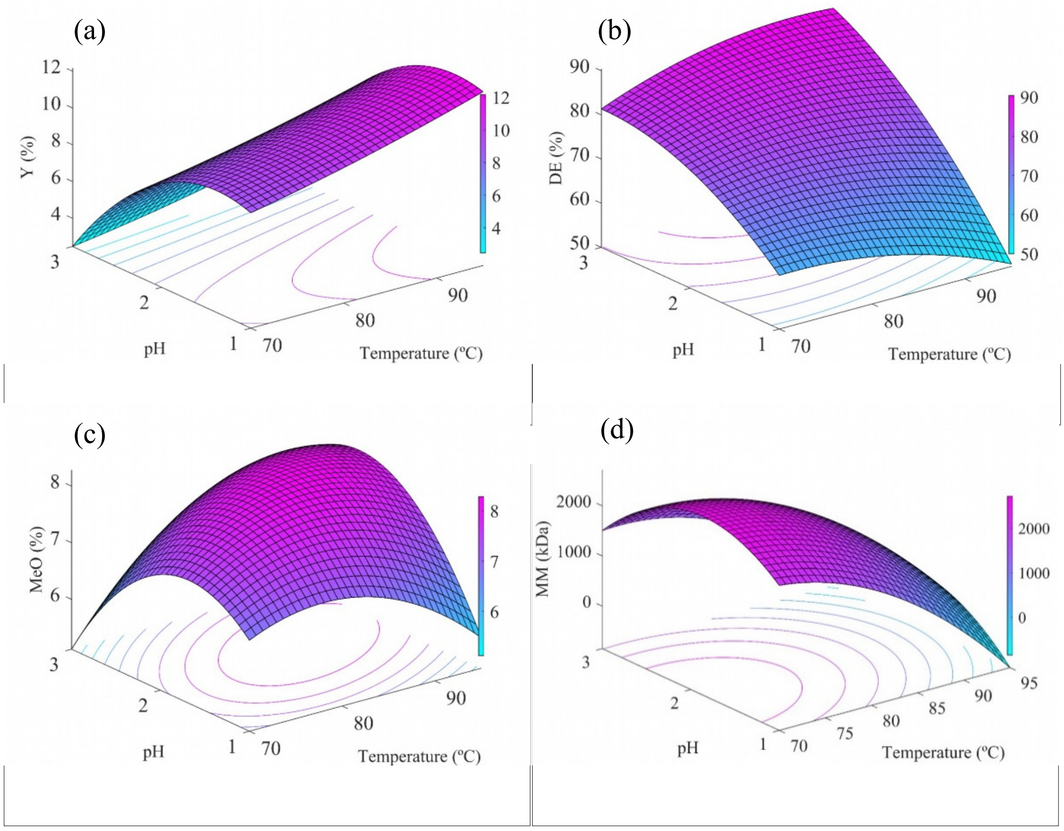

Figure S1. Response surface plot for the effects of temperature and pH with fixed time at 90 min on (a) yield (Y), (b) degree of esterification (DE), (c) methoxy content (MeO) and (d) molar mass (MM) (kDa), of pectin extracted.

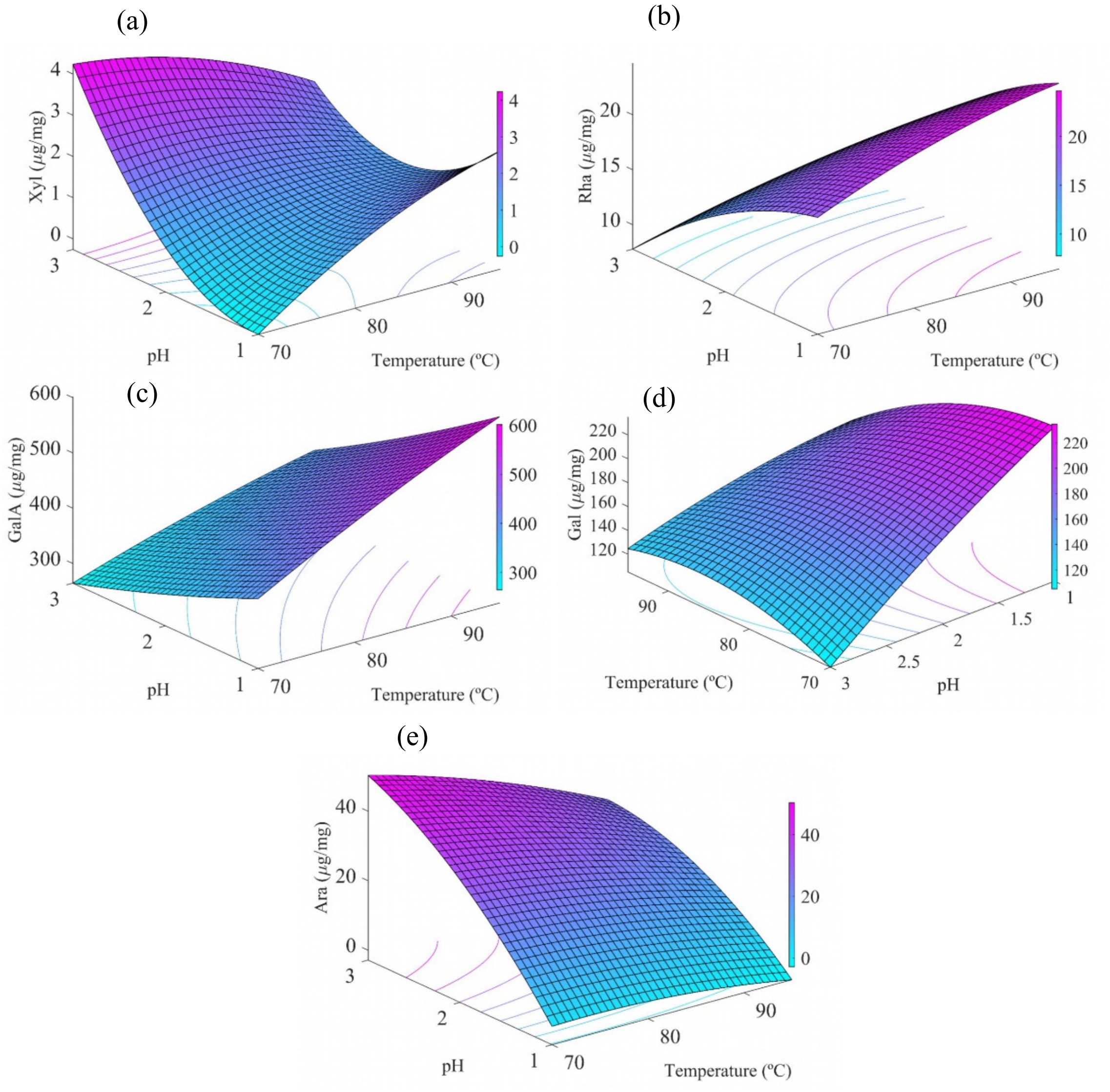

Figure S2. Response surface plot for the effects of temperature and pH with fixed time at 90 min on (a) xylose (Xyl), (b) rhamnose (Rha), (c) galacturonic acid (GalA), (d) galactose (Gal) and (e) arabinose (Ara) (μg/mg) content of pectin extracted.

Table S3. Molar mass (MM) at distinctive highest point, peak area and polydispersity index estimated with mathematical deconvolution calculated from the HPSEC runs evaluated.

	**Peak 1**			**Peak 2**			**Peak 3**		
**Run**	**MM (kDa)**	**Area %**	PDI	**MM (kDa)**	**Area %**	PDI	**MM (kDa)**	**Area %**	PDI
**1**	2348.4 ±65.24	64 ±2.8	8.5	19.94 ±0.42	36 ±2.8	1.6	0 ±0	0 ±0	0.0
**2**	1376.78 ±76.47	77 ±4.1	2.9	43.80 ±1.95	19 ±4.1	1.1	5.94 ±0.17	3.7 ±0.2	1.6
**3**	2687.38 ±77.45	75 ±0.3	1.8	59.90 ±1.51	21 ±0.1	2.6	5.54 ±0.22	4.2 ±0.3	1.0
**4**	1665.9 ±100.85	50 ±1.6	2.7	58.51 ±0.88	28 ±0.7	1.4	4.15 ±0.03	22.2 ±2	1.1
**5**	87.47 ±0.85	87 ±0.8	4.0	12.02 ±0.04	13 ±0.8	1.0	12.02 ±0.04	12.7 ±0.8	0.0
**6**	1708.41 ±44.34	29 ±1.8	2.9	58.83 ±0.15	37 ±2.7	1.5	5.48 ±0.02	33.7 ±1	1.0
**7**	1591.16 ±47.53	83 ±14.5	1.3	16.21 ±0.34	25 ±0	5.3	0 ±0	0 ±0	1.1
**8**	280 ±61.25	55 ±4.2	2.6	74.76 ±1.35	33 ±2.9	1.8	5.21 ±0.18	11.4 ±1.8	1.0
**9**	223.6 ±16.53	76 ±2.2	5.1	45.12 ±12.40	20 ±2.3	1.2	5.63 ±0.1	3.2 ±0.1	1.5
**10**	1148.72 ±114.91	7 ±11.4	1.8	198.79 ±4.47	73 ±11.3	4.0	14.22 ±0.11	20.5 ±0.3	1.1
**11**	2549 ±71.05	68 ±0.3	1.9	66.42 ±2.42	15 ±0.1	6.3	5.75 ±0.04	16.2 ±0.2	1.0
**12**	1157.93 ±65.51	58 ±0.8	2.9	63.31 ±1.28	27 ±0.6	1.7	4.94 ±0.07	15 ±0.8	1.0
**13**	2457.1 ±53.58	76 ±0.6	2.2	52.36 ±3.12	20 ±0.6	1.9	5.68 ±0.11	4.6 ±0.1	1.0
**14**	2678.84 ±66.55	78 ±0.3	1.9	55.52 ±2.15	22 ±0.3	9.5	0 ±0	0 ±0	1.0
**15**	2758.71 ±82.56	77 ±0.1	2.3	59.56 ±3.80	18 ±0.3	1.3	5.22 ±0.05	5 ±0.1	1.3
OP*	106.11 ±2.70	79 ±0.3	6.1	13.11 ±0.11	21 ±0.3	1.0	0 ±0	0 ±0	0.0
AP	339.4 ±11.4	100 ±0	8.9	0 ±0	0 ±0	0.0	0 ±0	0 ±0	0.0
CP	77.84 ±3.80	100 ±0	2.0	0 ±0	0 ±0	0.0	0 ±0	0 ±0	0.0

*Pectin extracted at optimum yield parameters (temp. 95 ºC, pH 1.35 and time 90 min)

Table S4. Comparison of experimental and predicted values of three additional representative extractions, varying parameters and their respective responses.

	70°C, 60 min, pH 1 (1)		82.5°C, 30 min, pH 3 (4)		82.5°C, 60 min, pH 2 (14)	
**Responses**	**Experimental value**	**Model value**	**Experimental value**	**Model value**	**Experimental value**	**Model value**
Y (%)	6.88	6.89	2.45	2.98	8.04	8.15
DE (%)	65.47	64.63	87.28	86.55	80.88	80.08
MeO (%)	6.87	7.10	6.66	6.58	9.00	8.43
AUA (%)	59.54	61.7325	43.22	43.05	63.16	59.78
MM (kDa)	2348.4	2275.30	1665.90	1607.56	2678.84	2631.55
GalA (µg/mg)	357.72	394.35	254.07	267.31	386.74	361.95
Ara (µg/mg)	9.09	7.68	47.31	47.72	32.82	33.64
Fuc (µg/mg)	3.44	3.23	2.93	2.79	3.54	3.37
Gal (µg/mg)	248.49	224.27	113.02	105.01	176.57	174.96
Xyl (µg/mg)	0	0.28	4.75	5.04	1.52	2.00
Rha (µg/mg)	15.71	17.37	7.45	7.62	16.70	17.02

References

Denman, L. J., & Morris, G. A. (2015). An experimental design approach to the chemical characterisation of pectin polysaccharides extracted from Cucumis melo Inodorus. *Carbohydrate Polymers*, *117*, 364–369. https://doi.org/10.1016/j.carbpol.2014.09.081
